# The Distinct Roles of Proximal and Distal Utility Values in Academic Behaviors: Future Time Perspective as a Moderator

**DOI:** 10.3389/fpsyg.2019.01061

**Published:** 2019-05-08

**Authors:** Juyeon Song, Yi Jiang

**Affiliations:** ^1^Department of Education, Brain and Motivation Research Institute (bMRI), Korea University, Seoul, South Korea; ^2^Department of Educational Psychology, Faculty of Education, East China Normal University, Shanghai, China

**Keywords:** utility value, effort cost, future time perspective, academic choice, procrastination

## Abstract

Utility value for long-term goals, named distal utility value, can be differentiated from utility value for short-term goals, named proximal utility value. The purposes of the present study were (1) to examine the distinct roles of proximal and distal utility value in predicting academic outcomes, (2) to test the mediating role of effort cost in the relationship between these two types of utility value and academic outcomes, and (3) to examine whether future time perspective moderates the role of distal utility value. The results from two independent studies provided compelling evidence for the distinct roles of proximal and distal utility value in predicting academic outcomes, as well as the mediating role of effort cost and the moderating role of future time perspective. Study 1, in which 598 Chinese students participated, demonstrated that proximal utility value negatively predicted effort cost, which in turn negatively predicted academic choice intentions. However, distal utility value did not predict effort cost but did directly predict academic choice intentions. Just as in Study 1, Study 2, in which 891 Korean students participated, found that proximal utility value negatively predicted avoidance intentions and procrastination, directly and indirectly, by lowering effort cost perception. By contrast, distal utility value positively predicted effort cost, which in turn positively predicted avoidance intentions and procrastination. Although distal utility value negatively predicted procrastination directly, the total effects of distal utility value on both academic behaviors were not significant. In Study 2, we also found that future time perspective moderated the relationship between distal utility value and effort cost. The findings of the present study extend the scope of expectancy-value theory, bridge expectancy and value theory with future time perspective theory, and provide guidelines for utility value intervention.

## Introduction

In the field of educational psychology, intervention studies on promoting academic motivation and achievement are increasingly being conducted to fill the gap between theory and practice (Hulleman and Barron, 2016). One simple utility value intervention program has been designed to increase students’ perceptions of the utility value of math or science based on expectancy-value theory (EVT). This program has been found to enhance students’ academic motivation and achievement (Hulleman and Harackiewicz, 2009). The effectiveness of this utility value intervention program has received much attention for its potential to promote motivation and achievement in the STEM fields, and it has been consistently verified across a variety of cultures and ages (Gaspard et al., 2015a; Hulleman et al., 2017; Shin et al., 2019). Although many utility value intervention programs are in the process of being developed, more empirical research on the specific role of utility value on educational outcomes is needed because it serves as the basis for developing such intervention programs.

Utility value is defined as the perception of how useful a particular task or activity is for achieving goals. Recent EVT studies have shown that utility value can be subdivided according to whether it applies to short- or long-term goals, and that individual students have different developmental tendencies for each type of utility value (Gaspard et al., 2017). When considering temporal discounting, utility values for short- and long-term goals can play different roles in academic motivation and behavior (Steel and König, 2006); however, researchers have primarily examined utility value in general. Furthermore, intervention programs have not clearly distinguished between the two types of utility value, making it unclear which type has the greater effect on academic motivation and behavior.

More specifically, students’ plans for their futures can profoundly impact their academic motivation (Husman and Lens, 1999; Oyserman et al., 2002). Students clearly recognize that an important purpose of education is to prepare them for the future. Hence, a student’s current motivation can be affected by future goals such as the desire to enter college or procure a good job. In fact, in past research, when students were asked their reasons for studying, the top-ranking answers were all related to future-oriented goals such as *to be admitted to a prestigious university, to make my dream come true, to get a better job or career*, and *to earn money* (Lee and Bong, 2016). Accordingly, it is necessary to identify the specific role of utility value for long-term goals (i.e., distal utility value) by comparing its role in various educational outcomes with that of utility value for short-term goals (i.e., proximal utility value).

To better understand of the role of distal utility value, it is necessary to comprehensively integrate EVT with future time perspective (FTP) theory because both theories consider future influences on students’ academic motivation (Husman and Lens, 1999; Wigfield and Cambria, 2010). In FTP theory, future time perspective is conceptualized as the general perception that individuals have regarding the future and is shown to exert an influence on students’ academic motivation (Oyserman et al., 1995; Malka and Covington, 2005; Pizzolato, 2006). Attempts to integrate EVT with other motivational theories have been made in a variety of areas, with a prime example being temporal motivation theory (TMT), which integrates EVT, temporal discounting, and need theory (Steel and König, 2006). However, studies on TMT have primarily been conducted in the fields of economics or business (Steel and König, 2006; Steel, 2007); they have rarely been conducted in an academic context.

Considering that time perspective is an important factor in explaining students’ academic motivation and behavior, we aimed to distinguish the respective roles of distal and proximal utility value in predicting academic outcomes. We also examined the potential moderating role of future time perspective on the role of distal utility value by integrating EVT and FTP theory.

## Theoretical Background

### Proximal Utility Value and Distal Utility Value

Expectancy and value theorists postulate expectancy for success and task value as the most direct predictors of students’ choice behaviors and achievement in academic settings (Eccles et al., 1983; Wigfield and Cambria, 2010). They have consistently found expectancy to be a stronger predictor for achievement than task value, whereas task value is a stronger predictor than expectancy for choice behavior (Eccles et al., 1983; Wigfield and Eccles, 2000; Dietrich et al., 2017). Utility value is identified with one of the task values together with interest (intrinsic value) and attainment value (Eccles et al., 1983), but it has received less attention than interest as an independent motivation construct. Recently, however, an increasing body of research on utility value has attracted more attention from motivation researchers to the role of utility value in academic motivation and performance (DeBacker and Nelson, 1999; Greene et al., 2004; Hulleman et al., 2010; Skaalvik et al., 2017).

Most previous research, however, has measured utility value as general utility value by combining utility value for short- and long-term goals (e.g., Chouinard et al., 2007) or has selectively assessed utility value either for short-term or long-term goals (e.g., DeBacker and Nelson, 1999; Greene et al., 2004). General utility value is a positive source of task value, which leads students to choose a task (Eccles et al., 1983). Thus, it is reasonable that general utility value should predict achievement motivation, effort, and classroom engagement (Chouinard et al., 2007; Kim et al., 2015; Doménech-Betoret et al., 2017). General utility value, however, sometimes does not predict students’ academic behaviors, such as behavioral and emotional risk, effort, and teacher-rated engagement, even though other motivational constructs, such as efficacy beliefs, attainment value, and cost have significantly contributed to predictions of these outcomes (Dever, 2016; Guo et al., 2016; Priess-Groben and Hyde, 2017).

The complex results regarding the role of utility value in academic behaviors may be due to the different definitions and measurements of utility value. Distal utility value refers to the perceived usefulness of tasks or activities for achieving long-term goals. For adolescents, examples of long-term goals are getting a good job, entering a prestigious school, or having a good future life; thus, they are related to the distal utility value of various school subjects. Proximal utility value, in contrast, refers to the perceived usefulness of tasks or activities for attaining short-term goals, such as applying learned knowledge to one’s daily life. Considering the distinct nature of long- and short-term goals, we expect that distal and proximal utility values function differently in students’ academic outcomes.

In particular, distal utility value predicts academic achievement, whereas its predictive paths for other academic outcomes have not been consistent (DeBacker and Nelson, 1999; Greene et al., 2004; Skaalvik et al., 2017). For example, distal utility value did not predict effort, persistence, help-seeking behavior, or anxiety (DeBacker and Nelson, 1999; Skaalvik et al., 2017), whereas it did significantly and positively predict strategy use and mastery goals (Greene et al., 2004). Although there are fewer studies that measure proximal utility value than distal utility value studies, recent intervention studies have demonstrated that proximal utility values increase both the interest and achievement of students (Hulleman and Harackiewicz, 2009). Therefore, proximal utility value can be expected to increase academic achievement behaviors, but the role of distal utility value remains unclear.

Most previous studies have examined the role of utility values in adaptive academic behaviors, such as effort, persistence, academic choice, and achievement (DeBacker and Nelson, 1999; Skaalvik et al., 2017). However, the different roles of proximal and distal utility values might be more pronounced for avoidance behaviors such as procrastination. According to the TMT, delayed behavior is fundamentally related to expectancy, value, temporal discounting, and the tendency to discount delayed rewards (Steel, 2007). In other words, the more people value a task and believe that they can do it successfully, the less likely they are to postpone the task. By contrast, people are more likely to delay their behavior when the reward for their actions is received in the distant future rather than in the near future. On the one hand, both proximal and distal utility values are considered value beliefs and thus can negatively predict procrastination. On the other hand, the role of these two types of utility value might vary as a function of the delay of reward. For example, students are not likely to delay studying to obtain better grades (reward) 1 day before an exam (the near future), whereas they might do so if the exam is in 3 weeks (the distant future). Therefore, proximal and distal utility value might differently predict procrastination in an academic setting. Nevertheless, to our knowledge, few researchers have examined both types of utility value simultaneously in a learning context.

### Effort Cost Perception as a Mediator

To better explain the differential roles of proximal and distal utility values, we should consider cost perception. For decades, EVT researchers have focused more on positive sources of task value, such as interest, utility value, and attainment value, which have been generally shown to yield approach motivation and behavior (Wigfield and Cambria, 2010). Recently, however, researchers have begun to place a new emphasis on the role of cost in understanding students’ academic functioning (Conley, 2012; Barron and Hulleman, 2015; Jiang et al., 2018). Cost is what is lost or invested in the process of completing a task or activity (Eccles et al., 1983). The perception of cost decreases the intention to engage in a task and increases the intention to quit (Eccles and Wigfield, 1995; Battle and Wigfield, 2003; Kurzban et al., 2013; Perez et al., 2014). In particular, increases in avoidance intention (e.g., drop-out intention) and avoidance behavior (e.g., procrastination) are unique consequences of cost (Perez et al., 2014; Jiang et al., 2018).

In psychological research, effort is considered a cost that can induce task aversiveness. Effort costs are more specifically considered a negative appraisal of the effort for a given task (Flake et al., 2015). Previous research has shown that task aversiveness, which is determined by such factors as required effort, time consuming, or task difficulty, is a typical task characteristic related to procrastination (Ferrari and Scher, 2000; Ackerman and Gross, 2005; Ferrari et al., 2006). Required effort, considered an index of effort cost, has been found to be an important predictor of procrastination, especially in academic tasks (e.g., Ferrari and Scher, 2000). Thus, when students perceive a class to have high effort costs (e.g., too much effort is required for the class), they tend to lose motivation for that class (Flake et al., 2015). Perez et al. (2014) also found that effort costs emerged as a significant predictor of the intention to leave STEM majors, whereas beliefs about competence, task value (consisting of interest, utility, and importance), opportunity cost, and psychological cost failed to predict such an intention. Collectively, effort cost is considered to play an important role in predicting avoidance intentions and behaviors, such as the intention to quit taking a course or procrastination.

Moreover, effort cost might function as a mediator between task value and academic outcomes. However, only a few studies have actually verified that task values can lower perceptions of effort cost, which in turn influence procrastination. In a recent experimental study, Song et al. (2016) demonstrated that task value (e.g., interest) plays a role in lowering individuals’ subjective perception of effort costs. More specifically, participants in the experimental group who performed an interesting task perceived the task to have lower effort costs than did those in the control group, even though participants in both groups had actually solved the same number of problems during the task. In other words, the intrinsic value of a task seems to act as an immediate reward for the effort people invest in the task. Thus, intrinsic value can make people perceive a task as having lower effort costs than the actual amount required for the task. This result was replicated in a survey by Song et al. (2016), in which interest was associated with reduced effort cost, which in turn decreased procrastination.

Interestingly, in the same study, Song et al. (2016) found that utility value increased perceived effort costs, which in turn positively predicted procrastination. Some previous studies have shown that utility value and cost are negatively correlated, whereas other studies have shown that they are positively correlated (e.g., Conley, 2012; Gaspard et al., 2015b). A positive correlation between utility value and effort cost would suggest that the student might have perceived that a valuable task that they feel is useful would require more effort. Students might also feel greater emotional burden and higher anxiety when participating in more important and useful tasks (Nie et al., 2011; Selkirk et al., 2011; Boehme et al., 2017). If proximal utility value and distal utility value are measured separately, however, we expect to find a different relationship between utility value and effort cost for each type of utility value. More specifically, utility value might increase effort cost only when it concerns long-term goals (i.e., distal utility value). We expect this because, much like interest, effort cost for highly valued tasks with proximal utility value will be compensated in the relatively near future, thereby precluding maladaptive academic outcomes to some extent. By contrast, when students perceive a task as useful for procuring a better job, they are only going to benefit from the task after a long period and only if they actually procure a job that requires the particular skills taught by that task. Thus, distal utility value might not decrease perceived effort cost, but rather might enhance it. Ultimately, effort cost might play a different mediating role for each type of utility value in predicting academic outcomes.

### Future Time Perspective as a Moderator

Expectancy-value theory researchers have continuously underscored the importance of understanding the relationship between utility value and future time perspective in deepening our knowledge of the role of utility value in students’ motivation (Wigfield and Eccles, 2002; Wigfield and Cambria, 2010). The domain-general perception of the future that we usually call *future time perspective* is clearly differentiated from distal utility value, which comprises the instrumental perception of a specific task (Husman et al., 2016). Future time perceptive is a trait-like individual tendency to envision the connection between the general future and the present, whereas perceived distal utility value is a context-specific instrumentality perception toward specific learning contexts or tasks (Husman et al., 2016).

As an individual difference, future time perspective can buffer the negative influence of distal utility value on academic functioning, such as increasing effort cost perception and procrastination and lowering intention to reengage in a task. Shechter et al. (2011) conducted an experimental study manipulating utility value and found cultural differences in the role of utility value in achievement behaviors. In particular, they distinguished proximal and distal utility values and compared the effects of two types of utility value. For East Asian participants particularly, distal utility value was more effective in increasing task interest and engagement than was proximal utility value. Conversely, for Western participants, emphasizing proximal utility value rather than distal utility value was more effective in task engagement and interest. The authors attributed these differences to cultural differences in the future time perspective. That is, East Asian participants may have been more motivated by distal utility value than proximal utility value because they could clearly visualize the connection between the new techniques acquired in the experiment and their long-term goals. This finding demonstrates that future time perspective can moderate the role of distal utility value, but no previous study has directly verified this. In fact, future time perspective is known to be closely related to academic delay of gratification (Bembenutty and Karabenick, 2004). The results of a meta-analysis similarly revealed that procrastinators are less likely to have a future time perspective (Sirois, 2014). Similarly, TMT also asserts that individual difference variables, such as future time perspective, might affect individuals’ sensitivity to rewards, and therefore should be considered as antecedents of procrastination together with task value and delay of rewards (Steel, 2007). Thus, in the current study, we directly examined whether future time perspective moderates the role of distal utility value in predicting effort cost perception and academic outcomes, including procrastination.

### Present Study

The present research aimed to explore the different roles played by proximal utility value and distal utility value in predicting effort cost and educational outcomes in a series of two independent studies. In Study 1, we examined the different roles of proximal utility value and distal utility value in predicting effort cost and academic choice intentions based on a cross-sectional data. For a subsequent study, Study 2, we collected an independent large longitudinal data, more suitable to test predictive relationships between variables. Specifically, we examined the distinctive roles of proximal utility value and distal utility value in predicting effort cost, avoidance intentions, and procrastination and further examined whether future time perspective moderates the effects of distal utility value on each outcome.

## Study 1

Students’ academic choices, such as course, major, and career choices, are the most representative academic outcomes that are explained by students’ perceptions of task value (Eccles et al., 1983). In Study 1, we examined the different roles of proximal and distal utility values in academic choice intentions. As the essential source of task value, both types of utility value could be expected to increase academic choice intentions. However, considering temporal proximity, proximal utility value could make stronger predictions than distal utility value.

We also tested the mediating role of effort cost in the relationships between utility values and academic choice intentions. We hypothesized that proximal utility value would strengthen academic choice intentions both directly and indirectly, by reducing effort cost perception. However, distal utility value might not significantly predict effort cost perception, thereby predicting academic choice intentions only directly. It is also possible that distal utility value might reduce academic choice intentions indirectly by increasing effort cost perception.

### Materials and Methods

#### Sample and Procedures

Participants were recruited from one school in Shanghai, China. The school is an academic track school, consisting of primary, junior, and senior high divisions. Students in this school largely come from middle-class family, which constitute the majority of the society. The present study focuses on middle and high school students. Eighteen classes were randomly selected and the sample comprises 598 adolescent students (318 boys, 272 girls, 8 did not indicate gender; 204 7th graders, 189 8th graders, 100 10th graders, and 105 11th graders; mean age = 14.8 years, *SD* = 1.61). Ninth and 12th grade students were prepared for school exams and did not participate in this study.

According to the guidelines of the East China Normal University’s institutional review board for human participants, the ethics approval is not mandatory for survey study that collected only students’ perceptions of academic motivation and without personal identifying information. Parental consent is not mandatory for this type of research according to the national regulations. However, parents were informed about this study through school announcement and no parents raised doubts about the study. A written informed consent form was given to all the students before the survey and they have free choice to decide whether to participate the study or not. Data were collected only from those who signed the consent form.

The survey was administered during regular class at the beginning of the school year. The students were informed that the questions on the survey would ask about their personal beliefs regarding different aspects of learning at school. They were also informed that the confidentiality of their responses on the survey would be strictly protected.

#### Measures

All survey items were written in Chinese and referred to the subject domain of mathematics. All survey items were based on a six-point Likert-type scale ranging from 1 (strongly disagree) to 6 (strongly agree). Items originally developed in English were put through a procedure of translation and back-translation as suggested by Brislin (1970). Cronbach’s alpha coefficients were used to examine the reliability of each measure.

##### Utility value

Two items on proximal utility value (e.g., Knowledge in mathematics comes in handy during everyday life and leisure time.) and four items on distal utility value (e.g., Subject knowledge in mathematics will be helpful for my future career.) were adopted from Gaspard et al. (2015b). The study demonstrated that the scales are reliable and valid, with reliability coefficients of α = 0.87 and 0.82 for proximal utility value and distal utility value, respectively.

##### Effort cost

Three items on effort cost (e.g., It takes too much effort for me to do well in Mathematics.) were adopted from Jiang et al. (2018). The reliability coefficient was α = 0.86. This scale had been used successfully in a prior study with different groups of adolescent students of varying ages (Jiang et al., 2016).

##### Academic choice intentions

Academic choice intentions were measured by three researcher-developed items measuring the degree to which students wanted to engage with math class and to choose math-related careers. The items were “If I could choose what class I want to take, I would like to choose a math class,” “I’d like to choose a math-related major at university,” and “I’d like to choose a math-related career.” The reliability coefficient was α = 0.89.

#### Data Analysis

All the analyses were conducted via Mplus 7.31. All items had relatively little missing data (less than 3.01%). We used the full information maximum likelihood approach in Mplus, which utilizes all available information when estimating the model parameters (Graham, 2009). Students were nested within classes, and the intraclass correlations (ICCs) ranged from 0.12 to 0.45. Because the multilevel structure was not of substantive interest, we conducted all analyses using the robust maximum likelihood estimator (MLR) and the design-based correction of standard errors (with type = complex) to account for any potential non-independence of data resulting from the nesting of students within classes (McNeish et al., 2017). The Supplementary Material provides specific input codes for the analysis.

Before testing our hypothetical model, we performed confirmatory factor analyses on the utility value scale to check its measurement properties. Then, a measurement model with all latent variables was examined to confirm the measurement properties and compute correlations among the latent variables. Structural equation modeling (SEM) was finally conducted to test the hypothetical model. Considering the possible influence of gender, we controlled for gender in the model (Gaspard et al., 2015b; Jiang et al., 2018). The chi-square statistics and multiple goodness-of-fit indexes, Tucker-Lewis Index (TLI), comparative fit index (CFI), root mean square error of approximation (RMSEA), and standardized root mean square residual (SRMR) were used to evaluate the overall model fit. For CFI and TLI, coefficients above 0.90 imply a satisfactory fit, while for RMSEA and SRMR, values under 0.05 indicate close approximate fit. Finally, values between 0.05 and 0.08 suggest a reasonable fit (Browne and Cudeck, 1993; Hu and Bentler, 1999).

### Results

Table 1 presents descriptive statistics and latent correlations. The moderate means and absolute values of skewness and kurtosis of less than 0.93 indicate that all measures produced an approximately normal distribution of scores based on the guideline of normality (i.e., skewness < 2, kurtosis < 7; West et al., 1995). All correlations among the variables were in the expected directions. Proximal and distal utility value were positively correlated with each other (*r* = 0.78), and were negatively correlated with effort cost (*r*s = -0.36 and -0.33, respectively). They were also positively correlated with academic choice intentions (both *r*s = 0.53). Effort cost was negatively correlated with academic choice intentions (*r* = -0.50).

**Table 1:** Descriptive statistics, reliabilities, and latent correlation coefficients in Study 1.

Variable	1	2	3	4	*M*	*SD*	Skew	Kurt	ICC	α
(1) Proximal utility value	–				4.10	1.34	-0.36	-0.67	0.29	0.87
(2) Distal utility value	0.78^∗^	–			4.60	1.14	-0.69	-0.14	0.25	0.82
(3) Effort cost	-0.36^∗^	-0.33^∗^	–		3.34	1.42	0.07	-0.93	0.12	0.86
(4) Academic choice intentions	0.53^∗^	0.53^∗^	-0.50^∗^	–	3.35	1.42	0.00	-0.89	0.13	0.89


*Skew, skewness; Kurt, kurtosis. ^∗^*p* < 0.001.*

#### Confirmatory Factor Analyses

The CFA results revealed a two-factor model for the items measuring utility value, with one factor representing proximal utility value and the other representing distal utility value [χ^2^(8, *N* = 598) = 17.352, CFI = 0.991, TLI = 0.995, RMSEA = 0.044, and SRMR = 0.016]. This two-factor model had considerably better model fit than did a single-factor model [χ^2^(9, *N* = 598) = 111.059, CFI = 0.947, TLI = 0.912, RMSEA = 0.138, and SRMR = 0.046]. These results suggest that proximal utility value and distal utility value are correlated but distinguishable.

#### SEM Analysis

Before performing an SEM analysis, we conducted a full CFA analysis with all latent variables and gender. The measurement model displayed a suitable fit: χ^2^(56, *N* = 598) = 157.840, CFI = 0.980, TLI = 0.972, RMSEA = 0.055, and SRMR = 0.038. All factor loadings were significant (*p* < 0.001), indicating that the latent variables were represented well by their respective indicators.

**FIGURE 1:**
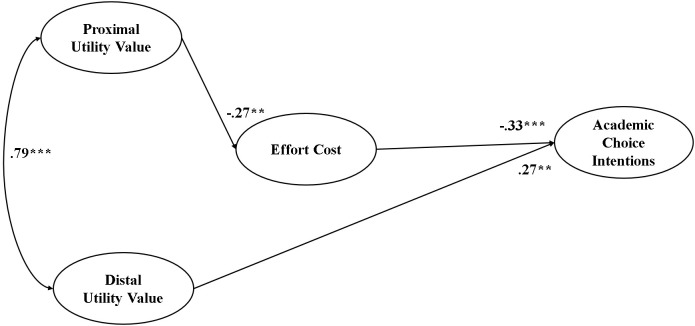
Statistically significant paths from the structural equation models in Study 1. Gender was included as a control variable in the model. Gender significantly predicted effort cost (β = 0.11, *p* = 0.004) and academic choice intentions (β = –0.15, *p* < 0.001), indicating that male students had lower effort cost perceptions and higher academic choice intentions than female students. ^∗∗^*p* < 0.01; ^∗∗∗^*p* < 0.001.

We then proceeded to test the paths among the latent variables via SEM. The fit for the hypothetical model was sufficient: χ^2^(58, *N* = 590) = 153.025, CFI = 0.980, TLI = 0.974, RMSEA = 0.053, and SRMR = 0.039. Figure 1 shows the statistically significant paths in this model (*p* < 0.05). Proximal utility value negatively predicted effort cost (β = -0.27, *p* = 0.005), which in turn negatively predicted academic choice intentions (β = -0.33, *p* < 0.001). Table 2 shows detailed information about the indirect paths in the model. As illustrated, proximal utility value positively predicted academic choice intentions (β = 0.09, *p* = 0.009) indirectly via effort cost. Conversely, distal utility value did not significantly predict effort cost, but it did directly and positively predict academic choice intentions (β = 0.27, *p* = 0.004).

**Table 2:** Total, direct, and indirect effects of indirect paths in Study 1.

Study 1													

Path					Total			Direct			Indirect		
			
					β	*SE*	*p*	β	*SE*	*p*	β	*SE*	*p*
Proximal utility value	→	Effort cost	→	Academic choice intentions	0.28	0.10	0.006	0.20	0.11	0.082	0.09	0.03	0.009
Distal utility value	→	Effort cost	→	Academic choice intentions	0.30	0.09	0.001	0.27	0.09	0.004	0.04	0.03	0.249


#### Summary

The findings of Study 1 support our hypotheses. First, the CFA revealed that proximal utility value and distal utility value correlated strongly with each other but clearly formed two independent factors. Moreover, as we hypothesized, these two types of utility value demonstrated distinct patterns in predicting effort cost and academic choice intentions. Proximal utility value lowered effort cost perception, thereby being able to positively predict academic choice intentions in an indirect way via effort cost. In other words, effort cost mediated the relationship between proximal utility value and academic choice intentions. In contrast, distal utility value emphasizes the benefits of achieving long-term goals, but it does not significantly predict effort cost. Nonetheless, distal utility value positively and directly predicted academic choice intentions.

## Study 2

The findings from Study 1 revealed that proximal and distal utility value could have distinct relationships with students’ academic motivation. However, Study 1 included only cross-sectional data. In Study 2, we examined the predictive ability of proximal and distal utility values using a longitudinal data set with different academic outcomes: avoidance intentions and procrastination. As mentioned in the introduction, the different roles of proximal and distal utility values might be more pronounced for avoidance-related outcomes such as avoidance intentions and procrastination. We also tested the mediating role of effort cost in the relationship between utility values and avoidance intentions and procrastination. We hypothesized that proximal utility value would negatively predict effort cost, but the predictive path from distal utility value to effort cost would be less clear. Study 1 found a non-significant path from distal utility value to effort cost, whereas previous research demonstrated a positive relationship between utility value and effort cost (Song et al., 2016). Therefore, we additionally tested future time perspective as a moderator to explain the inconsistent finding patterns regarding the relation between distal utility value and effort cost and to clarify the role of distal utility value in academic functioning.

### Materials and Methods

#### Sample and Procedures

Sample was collected from two middle schools and three high schools located in major cities including Busan, Daejeon, Ulsan, and Wonju, South Korea. The educational system of South Korea has 3 years of lower secondary school (middle school) and 3 years of upper secondary school (high school). In high school, there are two tracks: academic and vocational tracks. To ensure the samples are comparable across studies, we recruited students from academic track schools as in the Study 1. Thirty-eight classes were randomly selected and the sample comprises 897 adolescent students (195 boys, 691 girls, 11 did not indicate gender; 235 9th graders, 375 10th graders, 287 11th graders; mean age = 15.38 years, *SD* = 0.93).

According to the guidelines of the Korea University’s institutional review board for human participants, this study neither collected biomedical or behavioral data nor used questionnaire threats the rights and welfare of human participants, and thus IRB approval was not mandatory at the time of the survey. Parental consent was also not mandatory for this type of research; just like in China, parents were informed of the study through school announcements. All participants joined the study voluntarily.

In Study 2, we designed a longitudinal study to verify whether utility value precedes perceived effort cost and academic outcomes. Korean secondary schools begin their academic year in March, and a typical semester lasts for about 4 months. We thus measured utility value and future time perspective in the last week of March, the beginning of the semester (T1) and assessed effort cost, avoidance intentions, and procrastination 1 month later (T2). Both waves of surveys were administered during regular classes. Students’ were assured that their responses would never be disclosed to their parents or teachers and would be used only for research purposes.

#### Measures

All survey items were written in Korean and referred to the subject domain of English. Students responded to items on a six-point Likert-type scale ranging from 1 (strongly disagree) to 6 (strongly agree). As in Study 1, items that were originally developed in English were put run through a translation-and back-translation procedure (Brislin, 1970). The Cronbach’s alpha coefficients were used to examine the reliabilities of the measures.

##### Utility value (T1)

Items on utility value were developed based on Eccles and Wigfield’s (1995) scale. Originally, Eccles and Wigfield’s (1995) scale consists of only one item for each proximal and distal utility value. Based on Eccles and Wigfield’s (1995) conceptual definition, we developed one more item each for proximal utility value and distal utility value. Therefore, there are two items measuring distal utility values with a focus on how useful English is in future job and life (α = 0.79). The items are: “Studying English is useful for my future life” and “Studying English is useful for my future job and career.” Two other items measuring proximal utility value items focus on the usefulness of English in current daily life and academic life (α = 0.82). The items are: “Studying English is useful for my current life” and “Studying English helps my current daily life.”

##### Future time perspective (T1)

We adopted six *connectedness* items (e.g., What might happen in the long run should not be a big consideration in making decisions now.) from the short version (Hilpert et al., 2012) of the future time perspective scale (Husman and Shell, 2008). Connectedness is defined as a general tendency to make the connection between present activities or tasks and their influence on future goals (Husman and Shell, 2008). Connectedness needs to be clearly differentiated from the perception of distal utility value that comes from the instrumental view of a specific activity for a future goal (Husman and Shell, 2008), but may be strongly related to distal utility value. Previous researchers also investigated connectedness when they tested the role of the future time perspective in academic functioning or career decision-making (e.g., Husman et al., 2016; Adelman, 2017). The scale demonstrated a reliable Cronbach’s α coefficient of 0.75.

##### Effort cost (T2)

Items assessing effort cost were identical to those used in Study 1 except for the subject name. The reliability coefficient was α = 0.80.

##### Avoidance intentions (T2)

Avoidance intentions were measured by three researcher-developed items measuring the degree to which students did not want to engage with English learning. The reliability coefficient was α = 0.90. These items were: “I want to avoid an English-related career,” “I do not want to study English,” “I want to avoid studying English.”

##### Procrastination (T2)

Five procrastination items were adapted from the Melbourne Decision Making Questionnaire (Mann et al., 1997). The original items were developed to measure procrastination in decision-making and thus those were revised to assess procrastination, particularly in academic settings. The revised scale (e.g., “I delay studying math until it is too late.”) has already been used in previous studies and its measurement properties have been found to be reliable and valid (Jiang et al., 2018). In this study, the reliability coefficient was α = 0.90.

#### Data Analysis

All analyses were conducted in Mplus 7.31. As in Study 1, items had relatively little missing values (less than 8.14% for each item). The full information maximum likelihood estimation was used to handle the missing values. Although these data had a nested structure, their fairly low ICCs (ranging from 0.00 to 0.05) lead us not to perform design-based correction of standard errors. Before running our planned statistical models, CFAs were conducted to test the measurement properties of the utility value scale. A SEM analysis was performed to test the hypothetical model. Again, gender was included as a control variable in the SEM model. The Supplementary Material provides specific input codes for the analyses.

For the test of the interaction between distal utility value and future time perspective, we specified two models. In Model 1, we included all predictors in the SEM from Study 1 along with future time perspective. In Model 2, we included all terms from Model 1 along with a latent interaction term between distal utility value and future time perspective. To add to the robustness of the model, we also tested the latent interaction between proximal utility value and future time perspective in Model 3. To verify the interaction effect, we implemented a latent moderated structural equation modeling approach (LMS; Kelava et al., 2011), which is developed for the analysis of non-linear structural equation models with latent interactions (Kelava et al., 2011). Because model fit indices are insensitive to non-linear misspecifications (Mooijaart and Satorra, 2009), we were able to present model fit indices for Model 1 only and were unable to obtain standardized coefficients in Models 2 and 3. Despite these limitations, the LMS approach enhances power and reduced the likelihood of biased estimates by generating estimates of interactions that are unattenuated by measurement error (Maslowsky et al., 2015). To facilitate the interpretation of the LMS findings, we standardized all variables before conducting the analyses (Aiken and West, 1991). We determined the significance of indirect effect and evaluated the overall model fit with exactly the same rules that we used in Study 1.

### Results

Table 3 presents descriptive statistics and latent correlations. The moderate means and absolute values of skewness and kurtosis less than 0.82 indicate that all measures produced an approximately normal distribution of scores based on the guideline of normality (West et al., 1995). In terms of the correlations, proximal and distal utility values were positively correlated with each other (*r* = 0.78). Furthermore, both proximal and distal utility values were positively correlated with future time perspective (*r*s = 0.34 and 0.43, respectively), while they were negatively correlated with avoidance intentions (*r*s = -0.28 and -0.36) and procrastination (*r*s = -0.23 and -0.25). Future time perspective was negatively correlated with effort cost (*r* = -0.10), avoidance intentions (*r* = -0.45), and procrastination (*r* = -0.28).

**Table 3:** Descriptive statistics, reliabilities, and latent correlation coefficients in Study 2.

Variable	1	2	3	4	5	*M*	*SD*	Skew	Kurt	ICC	α
(1) Proximal utility value	–					4.07	1.15	-0.39	-0.08	0.01	0.82
(2) Distal utility value	0.78^∗∗^	–				4.70	1.06	-0.82	0.44	0.02	0.79
(3) Future time perspective	0.34^∗∗^	0.43^∗∗^	–			4.53	0.73	-0.21	0.29	0.01	0.75
(4) Effort cost	0.00	0.03	-0.10^∗^	–		4.03	1.08	-0.51	0.39	0.05	0.80
(5) Avoidance intentions	-0.28^∗∗^	-0.36^∗∗^	-0.45^∗∗^	0.46^∗∗^	–	3.54	1.23	0.09	-0.27	0.00	0.90
(6) Procrastination	-0.23^∗∗^	-0.25^∗∗^	-0.28^∗∗^	0.38^∗∗^	0.47^∗∗^	3.27	1.09	-0.03	-0.31	0.03	0.90


*Skew, skewness; Kurt, kurtosis. ^∗^*p* < 0.05; ^∗∗^*p* < 0.001.*

#### Confirmatory Factor Analyses

CFAs were conducted to ascertain the structure of the utility value items. In line with the results of Study 1, a two-factor model, with one factor representing proximal utility value and the other representing distal utility value [χ^2^(1, *N* = 848) = 2.241, CFI = 0.998, TLI = 0.990, RMSEA = 0.038, and SRMR = 0.005] had a much better model fit than did a single-factor model [χ^2^(2, *N* = 848) = 165.839, CFI = 0.786, TLI = 0.359, RMSEA = 0.311, and SRMR = 0.048]. These results indicate that proximal and distal utility values were independent constructs.

#### SEM Analysis

**FIGURE 2:**
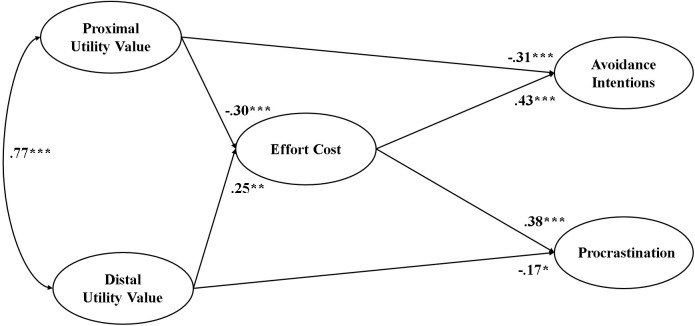
Statistically significant paths from the structural equation models in Study 2. The path coefficients from the SEM analysis, in which future time perspective was not included as either a predictor or a moderator. Gender was used as a control variable in the model. Gender positively predicted effort cost perception (β = 0.12, *p* = 0.002), meaning that male students had lower effort cost perceptions than did female students. However, gender did not significantly predict avoidance intentions or procrastination. ^∗^*p* < 0.05; ^∗∗^*p* < 0.01; ^∗∗∗^*p* < 0.001.

The measurement model displayed a suitable fit to the data: χ^2^(90, *N* = 897) = 400.386, CFI = 0.956, TLI = 0.941, RMSEA = 0.062, and SRMR = 0.052. All factor loadings were significant at *p* < 0.001, indicating that the latent variables were represented well by their respective indicators. We then performed a SEM analysis to test the hypothetical model before including the future time perspective in the model. The model fit was acceptable: χ^2^(92, *N* = 886) = 407.389, CFI = 0.955, TLI = 0.941, RMSEA = 0.062, SRMR = 0.054. Figure 2 shows the statistically significant paths in this model (*p* < 0.05). Proximal and distal utility values were positively correlated with each other (*r* = 0.77, *p* < 0.001), but their predictive paths to the dependent variables were different. As in Study 1, proximal utility value negatively predicted effort cost (β = -0.30, *p* < 0.001). Unlike in Study 1, in which we found no significant predictive path from distal utility value to effort cost, distal utility value positively predicted effort cost in the present study (β = 0.25, *p* = 0.003).

As for the outcome variables, proximal utility value negatively predicted avoidance intentions (β = -0.31, *p* < 0.001) while distal utility value negatively predicted procrastination (β = -0.17, *p* = 0.026). Effort cost positively predicted both avoidance intentions (β = 0.43, *p* < 0.001) and procrastination (β = 0.38, *p* < 0.001). Table 4 shows the indirect effects in the SEM model. Proximal utility value negatively predicted both avoidance intentions (β = -0.13, *p* < 0.001) and procrastination (β = -0.11, *p* = 0.001) via effort cost. In contrast, distal utility value positively predicted avoidance intentions (β = 0.11, *p* = 0.004) and procrastination (β = 0.09, *p* = 0.005) via effort cost.

**Table 4:** Total, direct, and indirect effects of the indirect paths in Study 2.

Path					Total			Direct			Indirect		
			
					β	*SE*	*p*	β	*SE*	*p*	β	*SE*	*p*
Proximal utility value	→	Effort cost	→	Avoidance intentions	-0.44	0.07	<0.001	-0.31	0.07	<0.001	-0.13	0.04	<0.001
Proximal utility value	→	Effort cost	→	Procrastination	-0.23	0.08	0.003	-0.12	0.08	0.123	-0.11	0.03	0.001
Distal utility value	→	Effort cost	→	Avoidance intentions	-0.02	0.08	0.800	-0.13	0.07	0.075	0.11	0.04	0.004
Distal utility value	→	Effort cost	→	Procrastination	-0.07	0.08	0.343	-0.17	0.08	0.026	0.09	0.03	0.005


#### Latent Moderated SEM (LMS) Analysis

**FIGURE 3:**
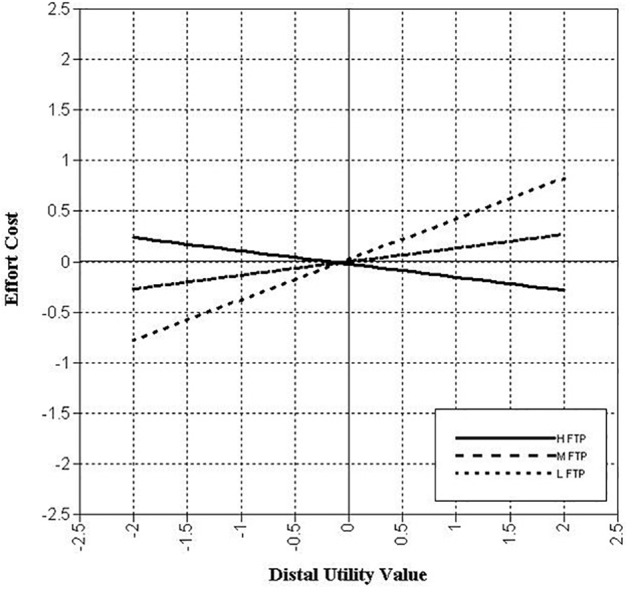
Plots of the moderating effect of future time perspective on the path from distal utility value to effort cost. H FTP = one standardized deviation above the mean of future time perspective; M FTP = the mean of future time perspective; L FTP = one standardized deviation below the mean of future time perspective.

LMS analysis was performed to test the potential interaction between two types of utility value and future time perspective. First, Model 1 included future time perspective as another predictor in the basic hypothetical model. The model fit was acceptable: χ^2^(192, *N* = 886) = 886.168, CFI = 0.918, TLI = 0.901, RMSEA = 0.064, and SRMR = 0.057. As Table 5 shows, future time perspective did not significantly predict effort cost, but did negatively predict avoidance intentions (*B* = -0.25, *p* < 0.001) and procrastination (*B* = -0.20, *p* = 0.002). We next examined Model 2, in which the interaction term between distal utility value and future time perspective was included as an additional predictor. The interaction between distal utility value and future time perspective on effort cost was statistically significant (*B* = -0.27, *p* = 0.019). As depicted in Figure 3, the interaction was such that the positive association between distal utility value and effort cost was weakened when students had a higher future time perspective. There were no significant interactions between distal utility value and future time perspective on avoidance intentions and procrastination. In the meantime, there was no interaction between proximal utility value and future time perspective on any dependent variable.

**Table 5:** Moderation effects of future time perspective in Study 2.

	Model 1				Model 2				Model 3			
			
	*R^2^*	*B*	*SE*	*p*	*R^2^*	*B*	*SE*	*p*	*R^2^*	*B*	*SE*	*p*
Effort cost	0.05				0.67				0.67			
Proximal utility value		-0.22	0.06	<0.001		-0.17	0.07	0.012		-0.20	0.07	0.003
Distal utility value		0.18	0.07	0.005		0.14	0.07	0.057		0.18	0.07	0.015
Future time perspective		-0.02	0.06	0.745		-0.02	0.07	0.751		-0.01	0.08	0.859
Distal UT × FTP						-0.27	0.11	0.019				
Proximal UT × FTP										-0.20	0.12	0.080
Gender: female		0.17	0.06	0.002		0.15	0.06	0.006		0.16	0.06	0.006
Avoidance intentions	0.41				0.52				0.53			
Proximal utility value		-0.32	0.08	<0.001		-0.33	0.10	0.001		-0.34	0.11	0.001
Distal utility value		-0.07	0.08	0.365		-0.07	0.10	0.493		-0.05	0.10	0.593
Future time perspective		-0.25	0.07	<0.001		-0.26	0.09	0.004		-0.27	0.09	0.005
Effort cost		0.64	0.06	<0.001		0.65	0.10	<0.001		0.65	0.10	<0.001
Distal UT × FTP						0.05	0.10	0.596				
Proximal UT × FTP										0.06	0.10	0.537
Gender: female		0.03	0.07	0.641		0.04	0.07	0.549		0.04	0.07	0.553
Procrastination	0.24				0.53				0.53			
Proximal utility value		-0.10	0.07	0.148		-0.10	0.08	0.227		-0.11	0.09	0.211
Distal utility value		-0.10	0.07	0.158		-0.11	0.08	0.188		-0.10	0.09	0.244
Future time perspective		-0.20	0.07	0.002		-0.20	0.08	0.015		-0.20	0.08	0.015
Effort cost		0.49	0.06	<0.001		-0.49	0.08	<0.001		0.49	0.08	<0.001
Distal UT × FTP						-0.03	0.11	0.797				
Proximal UT × FTP										-0.01	0.11	0.900
Gender: female		-0.01	0.06	0.831		-0.01	0.07	0.879		-0.01	0.07	0.869


*UT, utility value; FTP, future time perspective.*

#### Summary

As in Study 1, proximal utility value lowered effort cost perception. We also found that proximal utility value negatively predicted avoidance intentions and procrastination either directly or indirectly. Compared to Study 1, the most different result in Study 2 was that distal utility value positively predicted effort cost. As a result, the distal utility value of a task indirectly increased avoidance intentions and procrastination by enhancing perceived effort cost. We further found that these positive associations were moderated by future time perspective. Specifically, the positive relation between distal utility value and effort cost lessened as students had a higher future time perspective, which suggests a buffering role of the future time perspective on effort cost.

## General Discussion

The findings of the present study highlight the importance of differentiating between proximal utility value and distal utility value and the necessity of investigating effort cost and future time perspectives for improving our understanding of the role of utility value. We first found that adolescent students were able to distinguish between proximal and distal utility values. Moreover, proximal utility value and distal utility value were shown to have different functions in predicting effort cost perception and academic behaviors. Proximal utility value had an adaptive function in academic outcomes, whereas the role of distal utility value was inconsistent, particularly when predicting effort cost perception. Importantly, the mixed prediction was explained by the future time perspective.

### The Role of Proximal Utility Value

As hypothesized, proximal utility value negatively predicted effort cost perception in both Study 1 and Study 2 consistently, indicating that students perceived there to be less effort involved in studying mathematics or English when they perceived that mathematics or English were useful in daily life. By doing so, proximal utility value predicted academic behaviors not only directly but also indirectly by lowering the perception of effort cost. Specifically, as global task value (i.e., incorporating intrinsic and attainment value as well as utility value) is postulated as a positive predictor of academic choice intentions, behavioral engagement, and achievement in the expectancy-value model (Eccles et al., 1983; Dietrich et al., 2017), proximal utility value increased academic choice intentions in Study 1. Proximal utility value also worked as a prevent factor against avoidance intentions and procrastination.

The positive role of proximal utility value observed in the present study was similar to the positive role of interest proven in the existing literature. As interest reduced effort cost perception in a previous study (Song et al., 2016), proximal utility value negatively predicted effort cost across our study samples. In addition, interest is known to promote students’ academic engagement and to strengthen their intention to reengage in a task (Hidi and Renninger, 2006; Trautwein and Lüdtke, 2007; Reeve et al., 2015), and the present study demonstrated that proximal utility value had the same role. Both interest and proximal utility value may have acted as immediate rewards that offset effort cost, which may have played a role in lowering effort costs, avoidance intentions, and procrastination and in promoting choice intentions (Steel, 2007). In addition, interest and proximal utility value share the *relevance* concept as a key element. Students can perceive proximal utility value toward a certain task, when they find the task to be relevant in daily life (Hulleman and Harackiewicz, 2009). According to interest researchers, students can also find interest in this situation (Hidi and Renninger, 2006). Utility intervention research has indeed proved that connecting school subjects and daily life enhances students’ perception of utility value and, consequently, increases their interest (Hulleman et al., 2017). Therefore, it is necessary to test whether the positive role of proximal utility value is deeply linked to increase of interest.

### The Role of Distal Utility Value and Future Time Perspective

Contrary to proximal utility value, distal utility value did not negatively predict effort cost perception. Specifically, distal utility value did not have a significant prediction in Study 1. In Study 2, distal utility value even positively predicted effort cost, meaning that students can perceive more effort and time required to do a task when they perceive the task to be more useful for achieving *long-term* goals. This may be because the reward for the effort and time to be invested in the task for the long-term goal is not immediately accrued but delayed.

The relationship between distal utility value and effort cost can be tightly dependent on the perception of *time*. This idea seems to receive support from the moderating role of future time perspective. Study 2 demonstrated that the prediction of effort cost by distal utility value depended on levels of the future time perspective. In other words, the harmful influence of distal utility value on the perception of effort cost would be observed for students who have difficulty connecting their current behavior with the future consequences of it. Previous research has also found that the future time perspective can prevent the loss of ability belief after suffering a setback in an academic situation, such as receiving a poor grade (Adelman, 2017). Collectively, the future time perspective could at least play an important role in preventing loss of motivation.

In terms of prediction of outcomes, based on expectancy-value theory, distal utility value can be expected to predict academic choice intentions and as one of the task values (Eccles et al., 1983). Given the total effects of distal utility value, however, it only significantly predicted choice intentions but did not predict avoidance intentions or procrastination. Nevertheless, the moderation effect of future time perspective on the relationship between distal utility value and effort cost suggests the possibility that the future time perspective moderates the indirect prediction of distal utility value on avoidance intentions and procrastination from effort cost. In a meta-study, researcher found that future time perspective was negatively related to trait procrastination (Sirois, 2014). Findings from the present study further support that future time perspective, as a domain general personality, can moderate the role of domain-specific distal utility value in predicting procrastination in the academic domain. For adaptive functioning in an academic area, therefore, the future time perspective needs to be addressed together, particularly when educators emphasize the distal utility value of a task or activity.

### Theoretical Implications

The present research improves knowledge of the expectancy-value theory. Eccles et al. (1983) originally included both proximal and distal utility value in their definition of utility value, and some researchers have asserted that the role of future motivation should be more clearly identified (Husman and Lens, 1999; Wigfield and Cambria, 2010). Nevertheless, proximal and distal utility values have rarely been researched separately in a single study, and this has limited our understanding of the role of utility value. In this study, we distinguished between the two utility values, thereby enabling the construction of a clear understanding of the role of utility value in academic motivation and outcomes. In addition, recent researchers have begun to investigate cost as a motivational construct that is independent from task values; however, few studies have tested the relationship between task value and cost (Flake et al., 2015; Dietrich et al., 2017). Hence, this study has a theoretical significance in that it helps to elaborate the knowledge of the relationship between task value and cost by investigating effort cost as an independent construct.

Moreover, the present study bridges expectancy-value theory and future time perspective theory. Future time perspective researchers have continued to emphasize the influence of future time perspective on students’ academic motivation (Husman and Lens, 1999). Expectancy-value theory includes future motivation as part of utility value, and thus expectancy-value theorists have also suggested that future time perspective needs to be investigated together with utility value to attain a better understanding of the role of utility value (Wigfield and Cambria, 2010). Indeed, by verifying future time perspective as a moderator, we were able to better understand the role of utility value, particularly distal utility value.

In line with TMT, which integrates EVT and time-based theories such as picoeconomics or hyperbolic discounting (Steel and König, 2006), our studies successfully deepen our understanding of the role of utility value by integrating EVT and FTP theory in the academic setting. We used Eccles and colleagues’ EVT, which is distinguished from the classic expectancy × value theory by its distinction of the different sources of task value in achievement settings, including intrinsic value, utility value, and attainment value. In the same way that previous studies found that intrinsic value and utility value have different roles (Kim et al., 2015), our findings highlight the distinct roles of different types of utility value. All these findings suggest that value beliefs should be clearly specified to understand their role in motivation.

### Educational Implications

The results of the present study can provide more detailed guidelines for a utility value intervention in academic settings. Many recent researchers have been interested in an intervention centered on utility value and that will increase students’ motivation and performance (Gaspard et al., 2015a; Hulleman et al., 2017). They have succeeded in boosting the utility value of students with a simple method such as having students write about the relevance they find between course materials and daily life, which led to high interest and achievement. In those studies, the most key element of utility value was making the connection between course materials and students’ daily lives, which is more similar to proximal utility value (Hulleman et al., 2017). These intervention studies are significant in that they have provided a simple educational method to increase students’ interest and achievement.

However, it is important to be aware of what types of utility value should be generated. On the one hand, the present study partially supports the positive effect of utility value intervention. In particular, proximal utility value was beneficial for academic decisions and even lowered effort cost perception. The positive effect of proximal utility value intervention has been replicated by previous intervention studies (Hulleman et al., 2010; Gaspard et al., 2015a). Yet, despite the robustness of the positive effect of proximal utility value, it is unfortunate that, at least in Korea, students tend to perceive future-oriented values for studying more than present-oriented values (Lee and Bong, 2016). Consequently, educators need to help students discover and clearly internalize proximal values for studying.

On the other hand, this study demonstrated the possible limitation of a utility value intervention in terms of generating distal utility value alone. In this study, distal utility value works as a positive predictor for academic choice intentions but not for avoidance intentions or procrastinating behavior. Distal utility value even increased effort cost perception. Most previous intervention studies allowed participants to generate proximal utility value or both proximal and distal utility values rather than distal utility value alone (Hulleman and Harackiewicz, 2009; Gaspard et al., 2015a; Hulleman et al., 2017). This means the effectiveness of the distal utility value intervention has not yet been fully proven. Therefore, educators need to carefully use strategies to generate students’ distal utility value when encouraging their motivation and engagement. Considering the moderation effect of the future time perspective, having distal utility value can be effective only for students with a strong future time perception. Therefore, as previous studies have demonstrated, if students already study for the distal values from which they can gain, such as *for a prestigious university, future dream*, or *a better job or career*, educators need to strengthen students’ future time perspective, by, for example, explaining the influence of current behavior on the future.

### Limitation and Future Direction

Several limitations and future directions need to be addressed. First, this study was based on adolescents from limited number of schools. Although these are typical academic-track schools, they were not fully representative samples of Chinese and Korean students. Therefore, the generalizability of our findings to students in other school contexts requires further investigation. In addition, it will be interesting to explore whether our results are generalizable to Western cultures. Shechter et al. (2011) found cultural differences in the effect of distal and proximal utility value. In their study, distal utility value was more effective than was proximal utility value for East Asians, whereas proximal utility value was more beneficial for Westerners. Therefore, distal utility value might have an even more maladaptive role in academic motivation and engagement in Western cultures than in Eastern cultures. Potentially, the differences between two cultures could be explained by culturally encoded differences in the future time perspective. However, as we focused only East Asian samples, cross-cultural research is required to achieve a more universal understanding of the role and specific effect of utility value.

Second, the items for measuring the different types of utility value might need to be improved in future research. In the present study, the distal utility value items contained words representing the future, whereas the proximal utility value items measured in Study 1 did not directly refer to their current state. Although utility value in daily life is considered as a representative form of proximal utility value (Eccles and Wigfield, 1995; Shechter et al., 2011), scales for the proximal and distal utility values should be developed to more accurately define each utility value as a temporal distinction. Developing more precise scales to measure proximal and distal utility value will be helpful to better understand the role of these two constructs.

## Author Contributions

JS and YJ designed the research, collected the data of Study 1 and Study 2, and wrote the manuscript. JS analyzed the data.

## Conflict of Interest Statement

The authors declare that the research was conducted in the absence of any commercial or financial relationships that could be construed as a potential conflict of interest.
