# Supplementary material for: The Distinct Roles of Proximal and Distal Utility Values in Academic Behaviors: Future Time Perspective as a Moderator
Source: Front Psychol. 2019 May 8;10:1061. doi: 10.3389/fpsyg.2019.01061 (PMC6519296; doi:10.3389/fpsyg.2019.01061)
Supplement: Supplementary file 1 [file Data_Sheet_1.PDF]

Supplementary Material

1 Mplus Inputs

The variable names used in the analysis are as follows: putv = proximal utility value; dutv = distal utility value; efc = effort cost; ch = academic choice intentions, avnt = avoidance intentions; prc= Procrastination; ftp = future time perspective.

1.1 SEM analysis in Study 1

```
TITLE:      Study 1
DATA:       FILE IS utility_c_mplus.dat;
VARIABLE: NAMES ARE sid cntr sch grd cls nmb sex yy mm clus
                    putv1-putv2 dutv1-dutv4 efc1-efc3 ch1-ch3
                    putv dutv efc ch;
           Missing = ALL (-99);
           Usevariables = sex putv1-putv2 dutv1-dutv4
                    efc1-efc3 ch1-ch3;
           Cluster = clus;

ANALYSIS: TYPE = COMPLEX;

MODEL: putv BY putv1-putv2;
       dutv BY dutv1-dutv4;
       efc BY efc1-efc3;
       ch BY ch1-ch3;

       efc ON sex putv dutv;
       ch ON sex putv dutv efc;

MODEL INDIRECT:
    ch IND putv;
    ch IND dutv;


OUTPUT:    SAMPSTAT STANDARDIZED;
```

1.2 SEM analysis in study 2

```
TITLE:      Study 2 SEM
DATA:       FILE IS utility_k_mplus.dat;
VARIABLE: NAMES ARE sid sch grd cls nmb sex age sgc t1scr t2scr
                    t1ftp1-t1ftp6 t1putv1-t1putv2 t1dutv1-t1dutv2
                    t2efc1-t2efc3 t2avnt1-t2avnt3 t2prc1-t2prc5;
```

```
                    Missing = ALL (-99);
                    Usevariables = sex t1putv1-t1putv2 t1dutv1-t1dutv2 t2efc1-t2efc3
                                   t2avnt1-t2avnt3 t2prc1-t2prc5;

MODEL: putv BY t1putv1-t1putv2;
       dutv BY t1dutv1-t1dutv2;
       efc BY t2efc1-t2efc3;
       avnt BY t2avnt1-t2avnt3;
       prc BY t2prc1-t2prc5;

       efc ON sex putv dutv;
       avnt ON sex putv dutv efc;
       prc ON sex putv dutv efc;

 MODEL INDIRECT:
    avnt IND putv;
    prc IND putv;
    avnt IND dutv;
    prc IND dutv;

OUTPUT:     SAMPSTAT STANDARDIZED;
```

1.3 Latent moderated SEM (LMS) analysis in study 2

1.3.1 Model 1

```
TITLE:       Study 2 LMS MODEL1
DATA:        FILE IS utility_k_mplus.dat;
DEFINE:
STANDARDIZE t1ftp1-t1ftp6  t1putv1-t1putv2 t1dutv1-t1dutv2
                  t2efc1-t2efc3 t2avnt1-t2avnt3 t2prc1-t2prc5;
VARIABLE: NAMES ARE sid sch grd cls nmb sex age sgc t1scr t2scr
                            t1ftp1-t1ftp6 t1putv1-t1putv2 t1dutv1-t1dutv2
                            t2efc1-t2efc3 t2avnt1-t2avnt3 t2prc1-t2prc5;

                    Missing = ALL (-99);
                    Usevariables = sex t1ftp1-t1ftp6  t1putv1-t1putv2 t1dutv1-t1dutv2
                                    t2efc1-t2efc3 t2avnt1-t2avnt3 t2prc1-t2prc5;

MODEL: putv BY t1putv1-t1putv2;
       dutv BY t1dutv1-t1dutv2;
       ftp BY t1ftp1-t1ftp6;
       efc BY t2efc1-t2efc3;
       avnt BY t2avnt1-t2avnt3;
       prc BY t2prc1-t2prc5;
```

```
        efc ON sex putv dutv ftp;
        avnt ON sex putv dutv ftp efc;
        prc ON sex putv dutv ftp efc;

 MODEL INDIRECT:
    avnt IND putv;
    prc IND putv;
    avnt IND dutv;
    prc IND dutv;

OUTPUT:     SAMPSTAT STANDARDIZED;
```

1.3.2 Model 2

```
TITLE:          Study 2 LMS MODEL2
DATA:           FILE IS utility_k_mplus.dat;
STANDARDIZE t1ftp1-t1ftp6  t1putv1-t1putv2 t1dutv1-t1dutv2
                t2efc1-t2efc3 t2avnt1-t2avnt3 t2prc1-t2prc5;
VARIABLE: NAMES ARE sid sch grd cls nmb sex age sgc t1scr t2scr
                        t1ftp1-t1ftp6 t1putv1-t1putv2 t1dutv1-t1dutv2
                        t2efc1-t2efc3 t2avnt1-t2avnt3 t2prc1-t2prc5;

            Missing = ALL (-99);
            Usevariables = sex t1ftp1-t1ftp6  t1putv1-t1putv2 t1dutv1-t1dutv2
                        t2efc1-t2efc3 t2avnt1-t2avnt3 t2prc1-t2prc5;

ANALYSIS: TYPE = RANDOM;
     ALGORITHM=INTEGRATION;

MODEL: putv BY t1putv1-t1putv2;
       dutv BY t1dutv1-t1dutv2;
       ftp BY t1ftp1-t1ftp6;
       efc BY t2efc1-t2efc3;
       avnt BY t2avnt1-t2avnt3;
       prc BY t2prc1-t2prc5;

       efc ON sex putv dutv ftp;
       avnt ON sex putv dutv ftp efc;
       prc ON sex putv dutv ftp efc;

       dutvxftp | dutv XWITH ftp;
       efc ON dutvxftp;
       avnt ON dutvxftp;
       prc ON dutvxftp;

OUTPUT:     SAMPSTAT;
```

1.3.3 Model 3

```
TITLE:        Study 2 LMS MODEL3
DATA:         FILE IS utility_k_mplus.dat;
STANDARDIZE t1ftp1-t1ftp6  t1putv1-t1putv2 t1dutv1-t1dutv2
              t2efc1-t2efc3 t2avnt1-t2avnt3 t2prc1-t2prc5;
VARIABLE: NAMES ARE sid sch grd cls nmb sex age sgc t1scr t2scr
                    t1ftp1-t1ftp6 t1putv1-t1putv2 t1dutv1-t1dutv2
                    t2efc1-t2efc3 t2avnt1-t2avnt3 t2prc1-t2prc5;

           Missing = ALL (-99);
           Usevariables = sex t1ftp1-t1ftp6  t1putv1-t1putv2 t1dutv1-t1dutv2
                          t2efc1-t2efc3 t2avnt1-t2avnt3 t2prc1-t2prc5;

ANALYSIS: TYPE = RANDOM;
      ALGORITHM=INTEGRATION;

MODEL: putv BY t1putv1-t1putv2;
       dutv BY t1dutv1-t1dutv2;
       ftp BY t1ftp1-t1ftp6;
       efc BY t2efc1-t2efc3;
       avnt BY t2avnt1-t2avnt3;
       prc BY t2prc1-t2prc5;

       efc ON sex putv dutv ftp;
       avnt ON sex putv dutv ftp efc;
       prc ON sex putv dutv ftp efc;

      putvxftp | putv XWITH ftp;
      efc ON putvxftp;
      avnt ON putvxftp;
      prc ON putvxftp;

OUTPUT:    SAMPSTAT;
```
